# Phylogenetic Position of Avian Nocturnal and Diurnal Raptors

**DOI:** 10.1093/gbe/evu016

**Published:** 2014-01-21

**Authors:** Muhammad Tariq Mahmood, Patricia A. McLenachan, Gillian C. Gibb, David Penny

**Affiliations:** ^1^Instititue of Fundamental Sciences, Massey University, Palmerston North, New Zealand; ^2^Institute of Agriculture and the Environment, Massey University, Palmerston North, New Zealand

**Keywords:** raptor evolution, mitochondrial genomes, owls, Secretarybird, Accipitridae, Strigiformes

## Abstract

We report three new avian mitochondrial genomes, two from widely separated groups of owls and a falcon relative (the Secretarybird). We then report additional progress in resolving Neoavian relationships in that the two groups of owls do come together (it is not just long-branch attraction), and the Secretarybird is the deepest divergence on the Accipitridae lineage. This is now agreed between mitochondrial and nuclear sequences. There is no evidence for the monophyly of the combined three groups of raptors (owls, eagles, and falcons), and again this is agreed by nuclear and mitochondrial sequences. All three groups (owls, accipitrids [eagles], and falcons) do appear to be members of the “higher land birds,” and though there may not yet be full “consilience” between mitochondrial and nuclear sequences for the precise order of divergences of the eagles, falcons, and the owls, there is good progress on their relationships.

## Introduction

There has been good progress in resolving issues about the deeper relationships of modern birds. But that still leaves the major group of birds as Neoaves, and here there is less certainty about basic divisions. However, there has been some good progress and, for example, both McCormack et al. (2013) with nuclear data and Gibb et al. (2013) with mitochondrial (mt) data have proposed a general group of “water carnivores” that includes Pelecaniformes, Ciconiformes (including storks), and some related groups—but not the shorebirds (Charadriformes). Similarly, a group often called the “higher land birds” has been proposed (Johansson et al*.* 2001; Ericson et al. 2006) that is quite distinct from the water carnivores. This higher land bird group includes groups such as the songbirds, parrots, owls, falcons, eagles, Piciformes, and Coraciiformes.

Here, we are particularly interested in the group of “raptors,” both diurnal (e.g., falcons and eagles) and nocturnal (owls). Ideally, we would hope for “consilience” between nuclear sequences, mt sequences, and rare genomic changes (e.g., retroposons; Suh et al. 2011). The reasons why there is conflict and difficulty in resolution of the avian tree of life may be attributed to general issues such as taxon sampling, number of genes (size of data set), compositional bias, substitution saturation, and alignment issues (see Hernandez-Lopez et al. 2013; Kimball et al. 2013). Classical knowledge is often, but not always, right, for example, Cracraft (1981) included all raptors into Falconiformes, but more recently Hackett et al. (2008) proposed falconids as an order Falconiformes and they grouped accipitrids, cathartids, pandionid, and sagittarid into a separate order Accipitriformes. Similarly, the monophyletic status of some of the Neoavian orders remains uncertain (e.g., Gruiformes, Coraciiformes, Piciformes, and Falconiformes), and here again we would expect basic agreement for nuclear and mt data. Considerable progress has been made by using large data sets such as complete mtDNA genomes. It appears that the basal polytomy found in most early phylogenetic hypotheses proposed for Neoaves can be reduced by using complete mtDNA genomes and the phylogenetic signal can be improved by increasing the taxon sampling (Sorenson et al. 2003; Pereira and Baker 2006; Slack et al. 2007; Pratt et al. 2009; Gibb et al. 2013).

Cracraft (1981) reintroduced the idea of including owls in the Falconiformes, which was based on tarsometatarsal and pelvic morphology as shared with Pandionidae and Accipitridae (including falcons). His “division 3” included the Ciconiiformes and Falconiformes (including Strigiformes). But, Olson (1982) vigorously criticized Cracraft’s division that contains both flamingos and owls (combined) as monophyletic, because (according to Olson) it would be in vain to search for a morphological synapomorphy to define such a group within the class Aves. The classification by Amadon and Bull (1988) followed the traditional arrangement with all species of owls in the Strigiformes and Strigidae, which divided the strigids into Striginae (Typical owls) and Tytoninae (Barn owls, Bay owls).

More recently, Wink et al. (2009) included 120 taxa of the Strigidae and 23 taxa of the Tytonidae (the data set covers most of the genera) to study the phylogeny of owls (nocturnal raptors) based on cytochrome b and nuclear markers (LDH b intron DNA, RAG-1). This provides insight into the phylogeny and evolution of owls and the phylogenetic tree inferred from sequences of the cytochrome b gene, and nuclear RAG-1 was found to be generally in a good agreement with the classical taxonomy of owls (Sibley and Monroe 1990; Burton 1992; König et al. 1999; Weick 2006). The genetic data agreed with the attribution of species to a given genus with exceptions evident in the polyphyletic genus *Otus* and the paraphyletic *Bubo* complex.

It is expected that the two main owl lineages (barn owls and the ordinary owls) will come together. The two owl mt genomes currently available (*Tyto* and *Ninox*) do come together, but quite deeply, and could even be the result of “long-branch attraction (LBA)” (Hendy and Penny 1989). We support the suggestion (Pacheco et al. 2011) that additional complete mt genome sequences from deeply diverging Strigiformes are needed to discard the possibility of LBA; owls seem to have some of the highest rates of sequence evolution among Neoaves (Pratt et al. 2009). So, we expect that the availability of mt genomes for *Athene* and *Phodilus* will resolve fairly definitely that all the owls are monophyletic. Indeed, it would be surprising if the two groups of owls were not united. *Athene brama* (the Spotted Owlet) is expected to be deep on the same lineage as *Ninox novaeseelandiae* (an ordinary owl, see Wink et al. 2009), and *Athene* is expected to be the deepest divergence of common owls from *Ninox*. Similarly, *Phodilus badius* (the Oriental Bay Owl) should be about the deepest divergence with *Tyto alba* (the Barn Owl).

Regarding raptors, or birds of prey, there have been diverse opinions. One of our questions is whether the nocturnal raptors group (owls) joins together with either group of the diurnal raptors, together or combined (Falconidae and Accipitridae). There have been differences on this topic (see Hackett et al. 2008; Pacheco et al. 2011). Secretarybird (*Sagittarius serpentarius*) is predicted to lie deeper on the same combined lineage as Osprey (*Pandion haliaetus*) and the Accipitridae.

Recently, there have been an important attempt to integrate the phylogeny of Neoaves with biogeography (Ericson 2012), and this is paralleled by our recent attempt to integrate phylogeny and macroecology (Gibb et al. 2013). Without prejudging that the proposed phylogenetic groups are correct biogeographically, accipitrids, woodpeckers, and owls belong to Afroaves of Ericson (2012), whereas falconids belong to Australavis (though all are within higher land birds). So, an example of convergence in ecological adaptations would be the parallel evolution of diurnal predators in the two clades, “Accipitriformes” in Afroaves and “Falconiformes” in Australavis. This would predict that the falconids and accipitrids do not come together as a monophyletic group. Similarly, most striking are the parallels in lifestyle and behavior between the Secretarybird in Afroaves and the seriemas in Australavis.

There are several unresolved questions that we address; apart from the prediction that the two owls are monophyletic, as well as the Secretarybird grouping with Pandionidae and Accipitridae. We do not know yet whether the three raptor groups are monophyletic, though perhaps the consensus is now against their being so. So, we find that the owls are a natural group, and that the Secretarybird is deepest in Accipitridae. However, we find no good evidence that all raptors are monophyletic (unless there is reversion to nonraptor behavior in several groups).

## Results and Discussion

The three new mt genomes are sequenced and deposited in GenBank. The genomes are Secretarybird (*S. serpentarius*), GenBank accession number KF961184, 16,773 bp (complete); Oriental Bay Owl (*P. badius*), GenBank accession number KF961183, 17,086 bp (gap in control region [CR]); and the Spotted Owlet (*A. brama*), GenBank accession number KF961185, 16,194 bp (CR incomplete).

Oriental Bay Owl (*P. badius*) and Spotted Owlet (*A. brama*) follow the standard avian gene order which was first described in chicken (Desjardins and Morais 1990) and referred to as “ancestral avian” by Gibb et al. (2007). This gene order is consistent within Strigiformes. In contrast to other eagles and hawks (pandionid and accipitrids), Secretarybird (*S. serpentarius*) also has the ancestral avian gene order. Although it has been pointed out by Mindell et al. (1998) following all major avian phylogenies that avian gene order may have evolved independently several times, it will be interesting to see how gene order of seriemas compares with the falcons. This new information could be helpful to investigate parallel lifestyle and behavior between the Secretarybird and the seriemas (placed in Afroaves and in Australavis, respectively; see Ericson 2012).

**Fig. 1.—:**
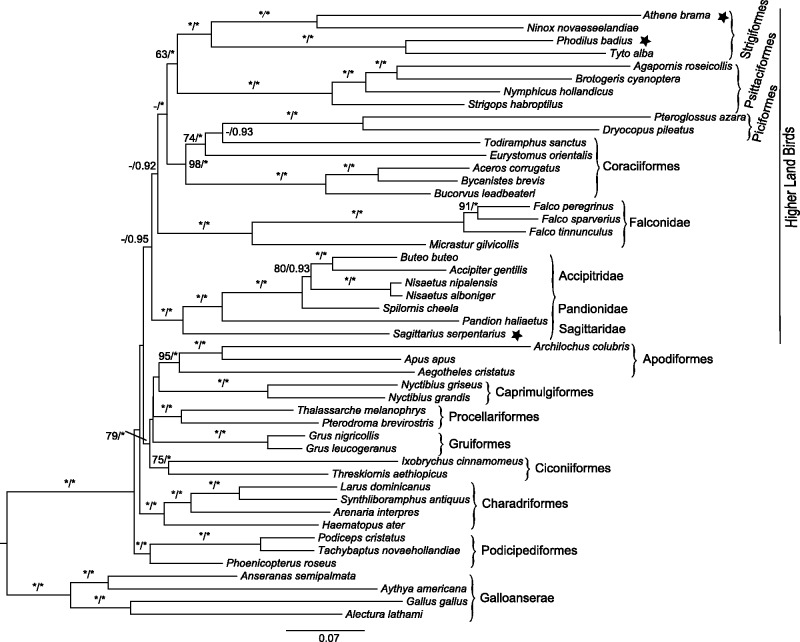
Rooted phylogram recovered from RAxML (using GTR + gamma + I). Five data partitions were used (third base position was RY-coded). New genomes reported in present investigation are marked with a star. Bootstrap and PP values are indicated for each node, with an * equaling 100% bootstrap support or a PP of 1.0 and – equaling less than 60% bootstrap or <0.9 PP. Values are not shown where both PP and bootstrap are less than 60%.

Our main approach to improve the raptor tree was the inclusion of additional taxa. Our main phylogenetic result is shown in figure 1 and is from a maximum likelihood (ML) analysis using GTR + gamma + I model using RAxML. As is our usual practice, the third codon position was RY coded. We have improved sampling within Strigiformes in order to avoid the possibility of LBA (the question raised by both Pratt et al. 2009 and Pacheco et al. 2011). This was a possibility, given the owls’ apparent high rate of evolution within Neoaves (Pratt et al. 2009). The two new mt genomes, of Oriental Bay Owl (*P. badius*) and Spotted Owlet (*A. brama*), represent Tytonidae and Strigidae, respectively. All four owls, the two common owls and the two barn owls, do come together on the tree; there appears to be no problem of LBA in this case. Presumably, this nocturnal group of raptors only evolved once within birds. This result is in agreement with the result of Hackett et al. (2008) based on nuclear sequences.

The next result was also as predicted; the Secretarybird is deepest on the Accipitridae lineage. There is a robust support for a clade that includes the families Accipitridae, Pandionidae, and Sagittaridae; thus, the Secretarybird (*S. serpentarius*) is deepest on the branch with Osprey (*Pandion haliaeetus*) and Accipitridae. This finding is in congruence with Lerner and Mindell (2005) and Hackett et al. (2008), although Wink and Sauer-Gürth (2004) (based on relatively short sequences) placed Secretarybird (*S. serpentarius*) with storks (Ciconiidae).

**Fig. 2.—:**
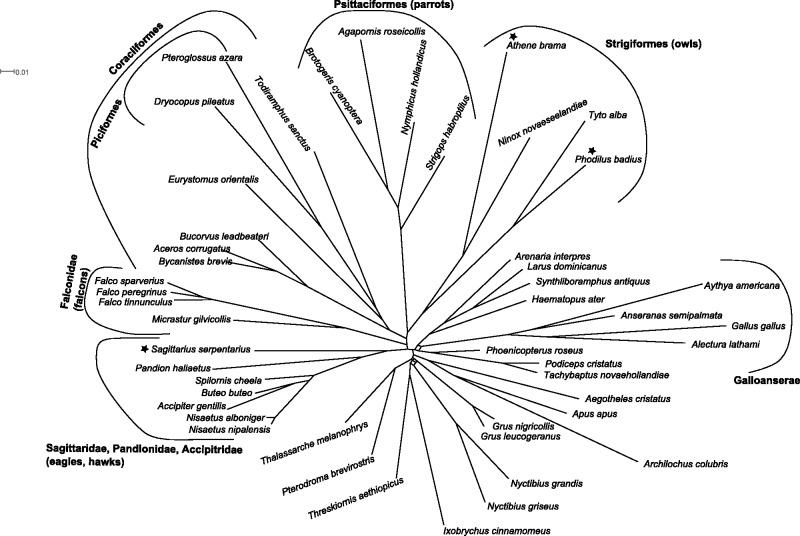
Consensus network of 48 Neoavian species based on analysis using MrBayes. New genomes reported in present investigation are marked with a star. Trees were sampled by Bayesian MCMC. Threshold = 0.2.

Although a morphological study (Livezey and Zusi 2007) recovered a monophyletic order Falconiformes consisting of five traditional families (Falconidae, Accipitridae, Pandionidae, Sagittaridae, and Cathartidae), none of the molecular studies that have included all five groups has found this relationship (Cracraft et al. 2004; Ericson et al. 2006; Hackett et al. 2008; Pacheco et al. 2011). We could not recover a sister relationship between Strigiformes and either or both of Falconidae and Accipitridae. This is consistent with previous molecular studies (Sibley and Ahlquist 1990; Gibb et al. 2007; Hackett et al. 2008; Pratt et al. 2009; Pacheco et al. 2011; McCormack et al. 2013), suggesting that such a relationship is not correct. Hackett et al. (2008) found that Falconidae was closely related to a clade of Passeriformes and Psittaciformes (parrots). A similar relationship of Falconidae and Passeriformes–Psittaciformes clade was recovered by some studies primarily based on nuclear introns (Wang et al. 2012) and retroposons (Suh et al. 2011). We did not find a Falconidae/Psittaciformes relationship in our analyses, neither with Bayesian (fig. 2) nor ML (fig. 1) analyses.

The phylogenetic analysis returned a relatively well-supported clade (posterior probability or PP = 1.0, ML = 63%) between Strigiformes (owls) and Psittaciformes (parrots), which had a good (PP = 1.0, ML = 58%) sister relationship with another well-supported clade (PP = 1.0, ML = 98%) between Piciformes (woodpeckers) and Coraciiformes (kingfishers). The addition of trogon (*Trogon viridis*) in the analysis slightly lowered the resolution. That could be partly because it forms an isolated long branch; therefore, it was excluded from most of our analyses (data not shown). At least one more deeply diverging mt genome within Trogoniformes would be helpful to improve the resolution of that particular node. However, we find that the bootstrap results are just small local changes in the underlying tree, and so the tree is “locally stable” in the sense of Cooper and Penny (1997). Our results, that Strigiformes and Psittaciformes are related groups, are in agreement with previous studies which recovered the same relationship (Sorenson et al. 2003; Harrison et al. 2004; Gibb et al. 2007; Brown et al. 2008; Wright et al. 2008; Pacheco et al. 2011). We present a resolved relationship of Strigiformes–Psittaciformes clade with Piciformes–Coraciiformes (referred to as SPPC henceforth) with support values PP = 1.0 and ML = 58%, which improved upon the support values (PP = 0.60–0.64, ML = 20%) reported by Pacheco et al. (2011) who found the same relationship. The inclusion of Passeriform taxa did not change the grouping of owls with parrots (data not shown). Nevertheless, and it is certainly significant that we also find a higher land group of birds, and that this includes all three groups of raptors.

Falconidae (falcons) appear to have shared a common ancestor with SPPC (PP = 0.92). Our ML analysis also found this relationship, although with poor support (ML = 25%). Accipitridae (hawks) were sister to the group comprising Falconidae and SPPC (PP = 0.95, ML = 26%) (fig. 1). We never recovered a direct sister relationship between accipitrids and falconids, which was in agreement with previous studies (Gibb et al. 2007; Slack et al. 2007). This finding (fig. 1) contrasts with Pacheco et al. (2011) who found a monophyletic relationship of diurnal raptors, which could be due to the choice of partitioning scheme (see Powell et al. 2013). Because Turkey Vulture (*Cathartes aura*) generated a long branch, it was removed after preliminary analysis (see Gibb et al. 2007; Slack et al. 2007). This could be partly because Cathartidae is possibly not a genuine raptor and its resemblance to Old World vultures is an example of convergent evolution (Tagliarini et al. 2009). Here again, additional sequences, including nuclear data, will be helpful.

Of the four nodes under investigation, the ML bootstrap values are quite low, that is (accipitrids and falconids–SPPC), (falconids and SPPC), (piciforms–coraciiforms and strigiforms–psittaciforms), and (strigiforms and psittaciforms). There are at least four possible explanations for the low resolution of ML tree. First, a “star tree paradox” (Steel and Matsen 2007) might also affect Bayesian methods when three or more lineages with high PP values (apparently resolved) diverge more or less simultaneously (Lewis et al. 2005; Kolaczkowski and Thornton 2006). Second, LBA might affect the Bayesian methods more than ML ones (Kolaczkowski and Thornton 2009). Third, Bayesian estimation of tree often gives quite high support values, which sometimes could be deceptively high (Douady et al. 2003). Finally, as compared with Bayesian methods, ML bootstrapping might underestimate the support values (Erixon et al. 2003). In a recent phylogenetic study on birds, McCormack et al. (2013) also observed weak ML support and high PP for some nodes when they used 416 locus data set, but ML support increased when they increased the size of their data set to 1,541 loci.

A consensus network of our Bayesian analysis (fig. 2) also suggested no close relationship between falconids and accipitrids. Further resolution of relationships between raptor groups would probably require additional complete mt genomes. In the present data set, Forest-Falcon (*Micrastur gilvicollis*) is the only representative of subfamily Polyborinae, among falconids. It appears to be a long branch. More representative mt genome sequence of this subfamily would be possibly helpful to discard the possibility of LBA problem. At least one more complete mt genome sequence from each of elanid kites (Elaninae), Old World vultures (Aegypiinae), and New World vultures (Cathartidae) would help resolve the relationship of cathartids, accipitrids, falconids, and SPPC within Neoaves.

Following the present tree, we find no good evidence that all raptors are monophyletic (unless there is reversion to nonraptor behavior in several groups). A recent description of a Middle Eocene skeleton (Mayr 2011) of a stem parrot (Pan-Psittaciformes) fossil *Messelastur gratulator* (Messelasturidae) may support such an argument, though it was previously considered to have closer affinities to either falconiform or strigiform birds (Mayr 2005). If future data on this fossil provide more affinities toward Psittaciformes, it would further support the idea that stem group parrots were predatory birds, but at present we cannot really support the idea.

## Conclusions

We are able to conclude here that owls (Strigiformes) are monophyletic, Secretarybird (*S. serpentarius*) forms a group with Accipitridae and Pandionidae, higher land birds are a natural group, and raptors are not a natural (monophyletic) group.

## Materials and Methods

### Taxon Sampling

The Secretarybird (*S. serpentarius*) was provided by Donna Dittman (Lousiana), sample number LSMUZ B-2458; the Oriental Bay Owl (*P. badius*) was supplied by Michael Wink from the Institute of Pharmacy and Molecular Biotechnology, Heidelberg, Germany, sample number 28304; and the Spotted Owlet (*A. brama*) was sampled by M.T.M. and was from Multan (south-west of Lahore) in Pakistan.

### Molecular Methods

Extractions of genomic DNA from each of the birds were performed at the Institute of Fundamental Sciences from 25 to 50 mg of muscle tissue using the High Pure PCR Template Preparation Kit (Roche Applied Science, Mannheim, Germany) according to the manufacturer’s instructions. To minimize the chance of obtaining nuclear copies of mt genes (NUMTs), 2–4 overlapping long-range polymerase chain reaction (PCR) fragments (3.5–12 kb in length) were first amplified using the Expand Long Template PCR System (Roche Applied Science). The products were excised from agarose gels and purified using a QIAquick Gel extraction kit (Qiagen GmbH, Hilden, Germany) as per the manufacturer’s instructions. These long-range products were subsequently used as template DNA for short-range PCRs to generate overlapping fragments 0.5–3 kb in length. Short-range primer combinations were found using our laboratory database as described in Slack et al. (2006), and any new primers required were designed using Geneious 5.5.7 (Drummond et al. 2011). Sequencing was performed using BigDye Terminator Cycle Sequencing reagents according to the manufacturer’s instructions (Applied Biosystems, Foster City, CA), and the reactions were run on an ABI 3730 automated sequencer (Applied Biosystems) by Massey Genome Service. Sequences were aligned using Sequencher 4.7 (Gene Codes Corp., Ann Arbor, MI) and then manually edited and checked for complete concurrence between overlapping sequences.

Where necessary (e.g., with length heteroplasmy in CRs from microsatellite repeats), PCR products were cloned using the TOPO TA cloning kit for sequencing (Invitrogen, Carlsbad, CA). For each region, at least three clones were sequenced to safeguard against PCR errors. In all cases, overlaps between sequences were sufficient to ensure synonymy and sequence identity was confirmed through Blast searches (http://www.ncbi.nlm.nih.gov/blast/), confirmation of amino acid translation in coding regions, and alignment with other species.

In addition to the 3 new bird mt genomes reported in this article, 47 other complete avian mt genomes from NCBI GenBank were included in the analyses: 43 Neoaves and 4 Galloanserae. Paleognath taxa were not included in this data set because their overall placement is now well established (Gibb et al. 2007; Slack et al. 2007). Instead, we rooted our Neoaves trees with the Galloanserae sequences (Gibb et al. 2007). We also repeated our analyses with six passerines included. The passerines do fall within the higher land birds, and their inclusion did not affect our conclusions of nonmonophyly of raptors (data not shown). The full data set is available from the authors on request.

### Phylogenetic Analysis

Sequences were aligned in Geneious 5.5.7 (Drummond et al. 2011) at the amino acid level for protein-coding genes and based on secondary structure for RNA genes (see Gibb et al. 2013). The data set has 12 protein-coding genes, 2 ribosomal RNAs (rRNA), and 22 transfer RNAs (tRNA). Gaps, ambiguous sites adjacent to gaps, NADH6 (light-strand encoded), and stop codons (often incomplete in the DNA sequence) were excluded from the alignment. The 12 protein-coding genes were separated into first-, second-, and third-codon positions (the third-codon position was RY-coded as explained by Gibb et al. 2007), whereas rRNA and tRNA genes were partitioned into stems (S) and loops (L), thus we use five data partitions (see Harrison et al. 2004). Protein-coding genes were checked for NUMTs by translating into amino acids.

A combined total of 13,430 nucleotides (excluding gaps) were used for analyses. We ran analyses in RAxML (Stamatakis et al. 2008) to carry out bootstrap replicates on the data sets where bootstrapping automatically stopped using the “majority rule” criterion. In addition to ML support, Bayesian posterior probabilities were also estimated. Bayesian analyses were carried out in MrBayes (Huelsenbeck and Ronquist 2001). Bayesian analyses were run for 10,000,000 generations with a burn-in value of 10%. Both RAxML and MrBayes were run using CIPRES Science Gateway (Miller et al. 2010). Trees were visualized in FigTree v1.4.0 (http://tree.bio.ed.ac.uk/software/figtree/ [accessed April 2013]). Consensus networks were implemented in SplitsTree version 4 (Huson and Bryant 2006).
